# Non-Contact Methods for Measuring Front Cavity Depths of Laboratory Standard Microphones Using a Depth-Measuring Microscope

**DOI:** 10.6028/jres.113.008

**Published:** 2008-04-01

**Authors:** Victor Nedzelnitsky, Randall P. Wagner

**Affiliations:** National Institute of Standards and Technology, Gaithersburg, MD 20899-8220

**Keywords:** acoustical calibration, calibration of microphones, depth-measuring microscope, front cavity depth, laboratory standard microphone, microphone calibration, microphone front cavity depth, reciprocity calibration, standard microphones

## Abstract

To achieve an acceptable degree of accuracy at high frequencies in some standardized methods for primary calibration of laboratory standard (LS) microphones, the front cavity depth *l_fc_* of each microphone must be known. This dimension must be measured using non-contact methods to prevent damage to the microphone diaphragm. The basic capabilities of an optical depth-measuring microscope were demonstrated by the agreement of its measurements within 0.7 μm of the known values of reference gage blocks. Using this microscope, two basic methods were applied to measure *l_fc_*. One (D) uses direct measurements at the microphone front surface annulus and conventional data reduction techniques. The other (GB) uses measurements at the surface of a gage block placed on the annulus, and plane-fitting data reduction techniques intended to reduce the effects of the slightly imperfect geometries of the microphones. The GB method was developed to provide a smoother surface of measurement than the relatively rough surface of the annulus, and to simulate the contact that occurs between the annulus and the smooth, plane surface of an acoustic coupler during microphone calibration. Using these methods, full data sets were obtained at 33 measurement positions (D), or 25 positions (GB). In addition, D and GB subsampling methods were applied by using subsamples of either the D or the GB full data sets. All these methods were applied to six LS microphones, three each of two different types. The GB subsampling methods are preferred for several reasons. The measurement results for *l_fc_* obtained by these methods agree well with those obtained by the GB method using the full data set. The expanded uncertainties of results from the GB subsampling methods are not very different from the expanded uncertainty of results from the GB method using the full data set, and are smaller than the expanded uncertainties of results from the D subsampling methods. Measurements of *l_fc_* using the GB subsampling method with only nine measurement positions exhibit expanded uncertainties (with coverage factor *k* = 2) within 4 μm, and can improve the uncertainty of microphone calibrations by an order of magnitude over the result from use of generic standardized microphone type nominal *l_fc_* values and tolerance limits.

## 1. Introduction

**Fig. 1:**
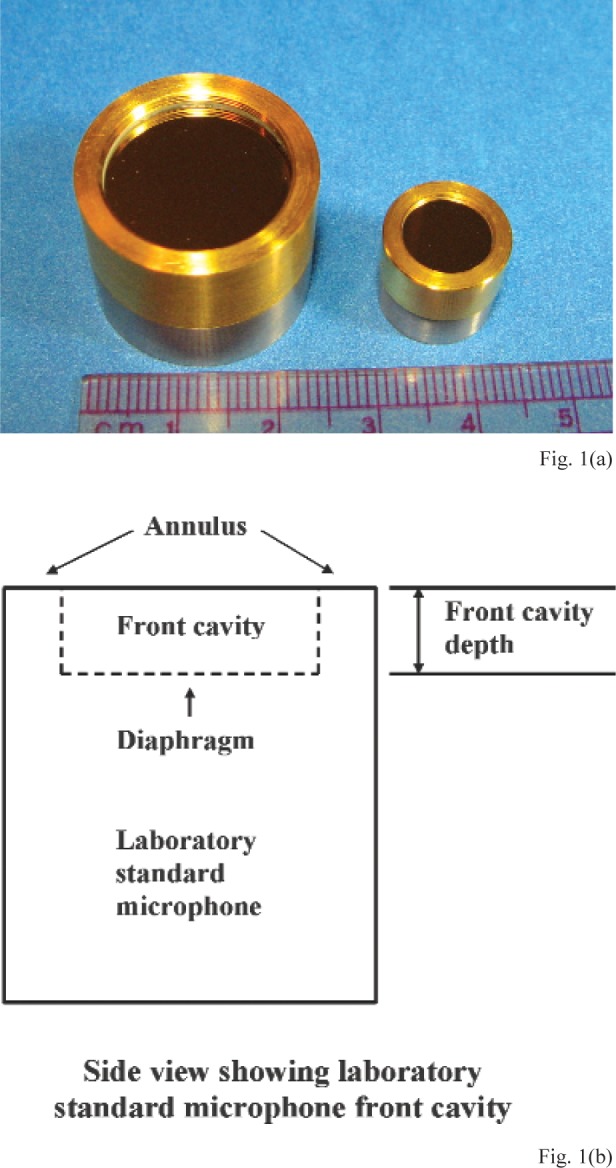
**(a)** Photograph of two types of laboratory standard microphones and ruler with 1 mm per fine division, **(b)** sketch showing laboratory standard microphone front cavity and the front cavity depth between the microphone annulus and diaphragm. (Not to scale)

**Fig. 2:**
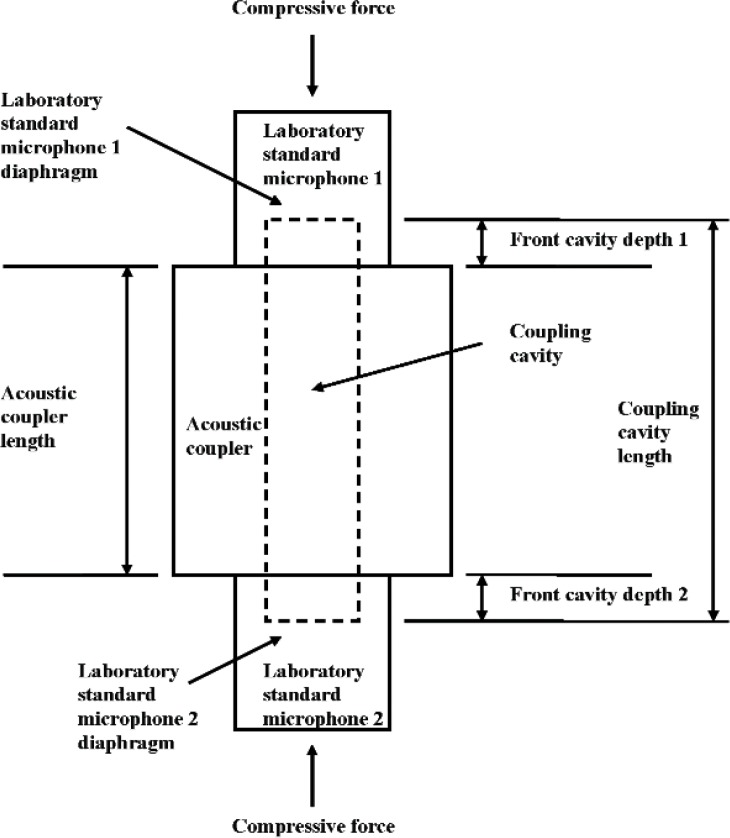
Block diagram of the equipment configuration that forms the coupling cavity used during plane-wave coupler microphone calibrations of laboratory standard microphones showing the relationship between the microphone front cavity depths and the coupling cavity length. (Not to scale)

Laboratory standard (LS) microphones are very stable capacitor microphones that are calibrated by primary methods and used as artifact reference standards to calibrate other LS microphones, measurement microphones, sound calibrators, and other acoustical instruments. Each type of LS microphone has standardized [1,2] electroacoustical properties and dimensions, with a circular diaphragm that is recessed from a front outer annular surface (herein called “annulus”). Figure 1a is a photograph of two different newer types of LS microphones. Figure 1b is a sketch that shows the front cavity depth (also known as the recess depth), which is the distance between the diaphragm of a LS microphone and the plane at the annulus. As shown in Fig. 2, the front cavity depths of LS microphones form a portion of the lengths of cylindrical plane-wave coupling cavities that can be used in certain standardized primary methods [3,4] for determining the pressure sensitivity of newer types of LS microphones.^1^ The annulus, which is very nearly parallel to the diaphragm, is placed in contact with one of the plane mating surfaces of a suitable acoustic coupler, so that the diaphragms and front cavity walls of a pair of LS microphones and the inner surface of the coupler are the boundaries of an essentially enclosed coupling cavity. A compressive force is applied to provide positive contact between the microphone annuli and mating surfaces of the acoustic coupler.

An important component of uncertainty in the pressure sensitivity levels^2^ determined by these methods is the uncertainty associated with the values of microphone front cavity depths used in determining the acoustic transfer impedance of the coupling cavity. Manufacturing variations in front cavity depths among individual LS microphones of a given type are significant, and nominal depth dimensions and permissible tolerance limits as given in Refs. [1,2] are 1.95 mm ± 0.1 mm for IEC Type LS1P microphones, and 0.5 mm ± 0.05 mm for IEC Type LS2aP microphones. If the front cavity depths of individual microphones are not measured, the use of these nominal dimensions and tolerance limits could lead to unacceptably large errors in determining the pressure sensitivity level at high frequencies. For example, for an air-filled plane-wave coupling cavity formed by the cavity of a coupler 4.7 mm long and the front cavities of two IEC Type LS2aP microphones, a total error of ± 0.1 mm in the sum of the front cavity depths would result in an error of ± 0.7 dB in microphone pressure sensitivity level at 25 kHz. This error alone would be at least 0.4 dB worse than the expanded uncertainty (with coverage factor *k* = 2) of primary calibration in pressure sensitivity level stated for this microphone type at this frequency by a number of national measurement laboratories in a recent international key comparison [5] of best measurement capabilities. To achieve an acceptably high degree of accuracy in calibration of LS microphones using air-filled plane-wave couplers, the front cavity depths of individual microphones must be measured.

Coupler length can be measured by conventional contact methods of dimensional metrology such as calibrated micrometers, optically encoded stylus probes, or coordinate measuring machines. However, measurement of microphone front cavity depth requires non-contact methods because the thin, highly tensioned metal diaphragm would be destroyed by conventional contact methods. A variety of techniques [5,6] have been used for measurement of LS microphone front cavity depth. Among these are versatile and convenient methods using an optical depth (*z*-axis) measuring microscope. The microscope is focused at positions on surfaces such as the microphone diaphragm, the annulus, or a gage block or optical parallel placed on the annulus. Focusing is performed with the microphone placed on an “*x*–*y*” plane perpendicular to the vertical *z*-axis, which corresponds to the line of microscope objective motion. An indicator coupled to the microscope focusing mechanism provides the *z*-axis coordinates of the measured positions at the surfaces. The front cavity depth is determined from differences between *z*-axis coordinates measured at different surfaces and from the thickness of the gage block or optical parallels (if used). For future work, computer-controlled optical scanning systems could be considered.

This paper investigates methods for measuring front cavity depth using an optical depth (*z*-axis) measuring microscope. Aspects of variations in method are investigated to enable good choices of procedural details for practical measurements that can be applied to calibration procedures in which the front cavity depth of each individual microphone is measured. In this context, good choices are those that are convenient, not excessively time consuming, and provide acceptable measurement results. Particular importance is attached to issues such as the effect of the compressive force (applied to the microphone and coupler mating surface) on front cavity depth, whether to perform measurements directly at the annulus or at a gage block placed on the annulus, spatial sampling (selection of number and positions of measurements) at different surfaces, data reduction methods, and the uncertainties in the measured results.

## 2. Equipment for Optical Measurement of Front Cavity Depth

Microphone front cavity depths were measured using a Nikon MM-11 measuring microscope system.^3^ This system includes a trinocular head, Nikon BD Plan bright-field/dark-field objectives with flat fields, BD Quadruple Nosepiece, Nikon Universal Epiilluminator system, and Nikon Digimicro digital micrometer system with SC-111 readout *z*-axis photoelectric digital measuring system with resolution of 0.5 μm. This system also includes a Nikon O3L measurement stage and Metronics Quadra-Chek 2000 readout *x*-axis and *y*-axis linear encoder digital measuring system with resolution of 1 μm. The trinocular head enables the use of visual observation through binocular eyepieces and video display modes by attaching a digital video camera with suitable controller, software, and display to the vertical photo tube of the trinocular head. To check the measurements performed by this microscope system, it is used to measure the thickness of a reference gage block, which has a hole through its center, at the top of a stack of gage blocks that were wrung together as needed.

The characteristics of the microscope objective used for the measurements reported here and by NIST in Ref. [5] are: magnification 40, numerical aperture 0.50, and working distance 9.8 mm. For the measurements reported in this paper, Nikon CFW 10x eyepieces were used with this objective so that the microscope magnification was 400. For both IEC Type LS1P and LS2aP microphones, the 9.8 mm working distance is sufficiently long to permit not only measurements at the diaphragm, but also at the portion of the diaphragm visible through a 6.7 mm diameter circular hole in a gage block 2 mm thick placed on the annulus. These working distances also permit measurements to be performed with a microphone and this gage block, held together in a spring-force fixture used to apply an adjustable, measured compressive force.

## 3. Data Acquisition Procedures

On microscopic examination, the annulus of a LS microphone often is seen to include annular or partially annular marks of high points (ridges) and low points (valleys). When the microphone is clamped against a smooth plane surface of a coupler, contact between this plane surface and the annulus should be modeled to occur at the high points of this annulus. The field of view of the microscope at usefully high magnification is only a small part of the annulus, so that even many direct measurements at various positions on the annulus represent only a very limited spatial sampling that does not necessarily include the high points of the annulus that define the plane of contact between the annulus and coupler surface. In this sense, the measurement of front cavity depth that relies on direct measurements at the annulus is based on an inadequate model of the contact between the annulus and the smooth plane surface of an acoustical coupler, and leads to an inadequate measurement that tends to underestimate the front cavity depth to be added to the coupler length during calibration. To avoid this problem, a gage block of known thickness, which has relatively smooth and parallel upper and lower planar surfaces, is placed on the annulus to perform the measurement. The contact between the annulus and the block offers a better model of the contact between the annulus and the smooth plane surface of an acoustical coupler.

Front cavity depth measurements of two IEC Type LS2aP microphones were performed at NIST as part of the CCAUV.A-K3 key comparison on the sensitivity calibration of such microphones [5]. The front cavity depths were determined by placing a gage block of calibrated thickness on the annulus, focusing at the upper surface of the block, and focusing at the visible portion of the microphone diaphragm through a circular hole in the block.

For each microphone, bright-field and dark-field measurements were performed and the average of all these measurements was taken. However, no systematic difference was observed between the bright-field and the dark-field measurements. That and subsequent experience also indicated that for very clean and bright surfaces such as particularly clean and reflective microphone diaphragms, it was easier to perform dark-field measurements. Therefore, decisions made at NIST to use bright-field or dark-field observation for a given surface now are based on the amount of detail observed and the perceived ease with which focal plane position can be determined.

To avoid uncertainties in focal plane position due to adaptation of the eyes, a video display was used instead of the eyepieces for the NIST front cavity depth measurements described in Ref. [5]. Experience at NIST subsequent to these measurements indicated that the limitations from video display resolution (due to limited numbers of pixels in the image) and video frame processing update rates could be comparable to or worse than the limitations created by the adaptation of the eyes during observation via the eyepieces. This was particularly so if observations via the eyepieces were made relatively quickly by focusing up and down to obtain the center position of sharpest focus. Measurements by such observation via the eyepieces are convenient and rapid, and seemed to minimize the effects of adaptation of the eyes. Therefore, the measurements reported here have been obtained by observation via the eyepieces.

Front cavity depth measurement data were acquired with the microscope on six microphones, three of IEC Type LS1P and three of IEC Type LS2aP. Using this microscope, two basic methods were applied to measure the front cavity depth of each microphone. One, which is called here the direct (D) method, is performed by focusing at the diaphragm and the annulus directly and applying conventional data reduction techniques (described in Sec. 4). The other, which is called here the gage block (GB) method, uses data acquired by focusing at the upper surface of a gage block placed on top of the annulus and by focusing at the central portion of the diaphragm visible through the hole in the center of the gage block. The GB method uses plane-fitting data reduction techniques (described in Sec. 4) intended to reduce the effects of the slightly imperfect geometries of the microphones. Dark-field viewing was used for the diaphragm in both methods and for the gage block in the GB method. Bright-field viewing was used for the annulus in the D method.

**Fig. 3:**
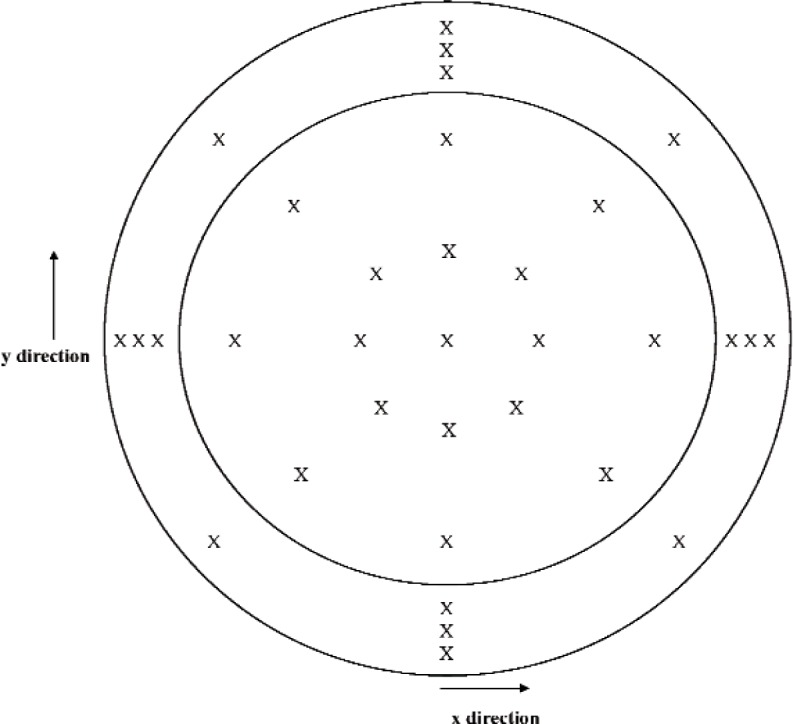
Approximate positions (indicated by an “X”) at the microphone annulus and diaphragm where data were acquired for the direct (D) method. (Not to scale)

For each set of measurements performed using the D method, data were acquired at seventeen positions on the diaphragm, and at sixteen positions on the annulus. The nominal *x*-axis and *y*-axis coordinates of these positions are given in Table 1 for IEC Type LS1P microphones, and in Table 2 for IEC Type LS2aP microphones. For each set of measurements, the first *z*-axis coordinate measurement was performed at the center of the diaphragm, which is the coordinate system origin. After data were acquired at all positions at the diaphragm and the annulus, the measurement at the diaphragm center was repeated. Figure 3, which applies to the direct measurements for both microphone types, shows the approximate positions measured at the diaphragm and the annulus.

**Fig. 4:**
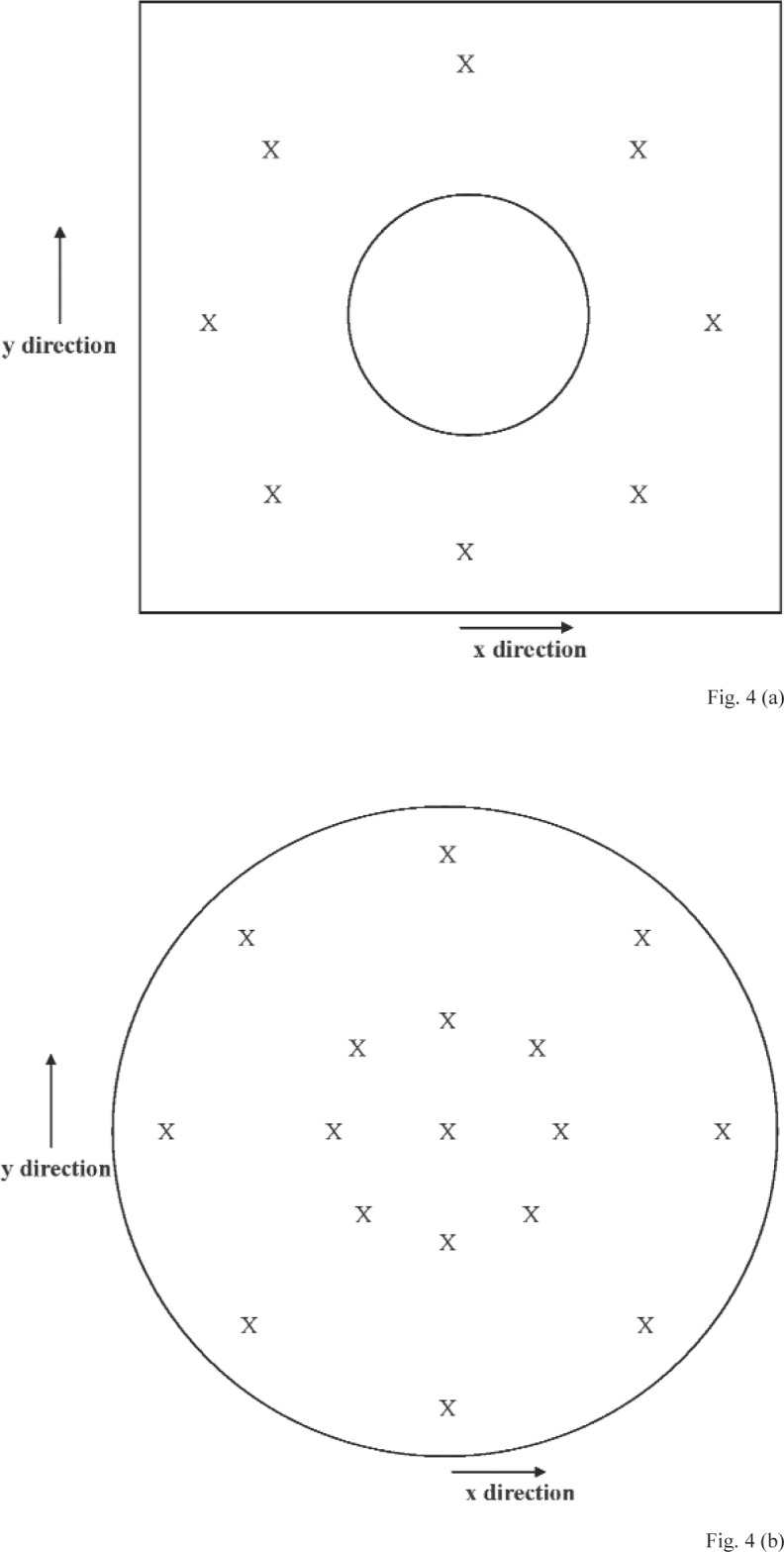
Approximate positions (indicated by an “X”) at the **(a)** gage block and **(b)** microphone diaphragm (portion viewed through the hole in the center of the gage block) where data were acquired for the gage block (GB) method. (Not to scale)

For each set of measurements performed using the GB method, data were acquired at seventeen positions at the diaphragm, and at eight positions at the gage block. The nominal *x*–*y* coordinates of these positions, which apply to both microphone types, are given in Table 3. For each set of measurements, the first *z*-axis coordinate measurement was performed at the center of the diaphragm, which is the coordinate system origin. After data were acquired at all positions at the diaphragm and the gage block, the measurement at the diaphragm center was repeated. Figure 4a shows the approximate positions measured at the gage block surface, and Fig. 4b shows the approximate positions measured at the diaphragm portion viewed through the hole in the gage block.

To determine the effect on the front cavity depth of the compressive force applied during microphone calibration using plane-wave couplers, a mechanical spring-force fixture, comprising horizontal top and bottom plates, was constructed to replicate this force during the optical determination of the front cavity depth. The microphone was placed on a disc-shaped adapter that fits on top of a washer-shaped load cell, which is set on the bottom plate and measures the force. A gage block was then mounted on top of the annulus so that the hole in the block was centered over the microphone diaphragm. The top plate was then positioned on top of the gage block. This plate has a 24 mm diameter hole in the center that allows viewing the microphone diaphragm and most of the top surface of the gage block. To provide and to adjust the compressive force, two screws that protrude downward through holes in the top plate were threaded into standoffs mounted vertically on the bottom plate. Wave washers placed between the screw heads and the top plate act as springs to help control the amount of force applied as the screws are tightened incrementally by alternating between screws.

For each microphone, the front cavity depth was measured with the microphone mounted in the spring-force fixture with a 13.3 N compressive force applied, which corresponds to the maximum compressive force currently used at NIST during calibrations. Before the microphone was removed from the fixture, the measurement was repeated with the screws loosened so that only the 0.4 N force due to the weight of the top plate was applied to the gage block and microphone. For one microphone of each type, an additional measurement was made with the top plate removed, but with the gage block and microphone remaining in place on top of the adapter, load cell and bottom plate.

## 4. Data Reduction Procedures

Front cavity depths were calculated from the full data sets obtained using the D method by subtracting the mean *z*-axis coordinate of all the positions measured at the diaphragm from the mean *z*-axis coordinate of all the positions measured at the annulus. Additional calculations were done with subsamples of the full data set by utilizing general techniques that have been implemented by various laboratories and described in other documents [5,6].

**Fig. 5:**
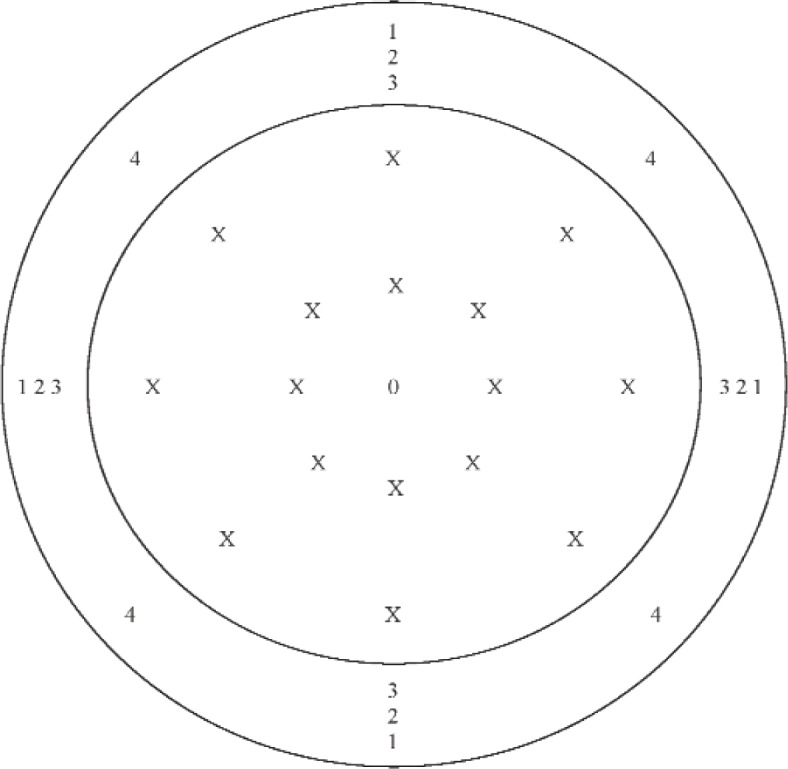
Approximate positions at the microphone annulus and diaphragm where data were acquired for direct measurement subsampling method A (DSMA). All positions at the annulus shown with a given number were included in the same subset. The *x*–*y* origin at the center of the diaphragm, which is indicated by an “O”, was included in every diaphragm subset. An “X” indicates positions from the full data set that were not included in any DSMA subset. (Not to scale)

**Fig. 6:**
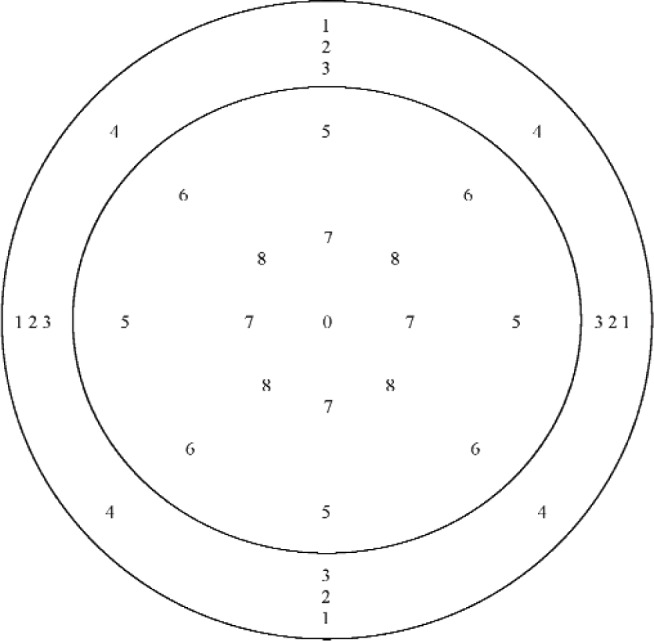
Approximate positions at the microphone annulus and diaphragm where data were acquired for direct measurement subsampling method B (DSMB). All positions at the diaphragm and the annulus shown with a given number were included in the same subset. The *x*–*y* origin at the center of the diaphragm, which is indicated by an “O”, was included in every diaphragm subset. (Not to scale)

One method determines the front cavity depth from subsampled data by subtracting the measured *z*-axis coordinate of the position at the origin, which is at the center of the diaphragm, from the mean *z*-axis coordinate of four positions measured at the annulus. Four different subsets were used to calculate four different front cavity depths. This method will be referred to as direct measurement subsampling method A (DSMA). The measurement positions included in the various subsets are indicated in Fig. 5. All positions at the annulus shown with a given number were included in the same subset. An “O” indicates the origin at the center of the diaphragm, and an “X” indicates positions from the full data set that were not included in any DSMA subset. The second method determines the front cavity depth from subsampled data by subtracting the mean *z*-axis coordinate of five positions measured at the diaphragm, including the origin, from the mean *z*-axis coordinate of four positions measured at the annulus. This method will be referred to as direct measurement subsampling method B (DSMB). Four different subsets were used for each surface to calculate eight different mean *z*-axis coordinates, four for the diaphragm and four for the annulus. The measurement positions included in the various subsets are indicated in Fig. 6. All positions at the diaphragm shown with a given number were included in the same subset for the diaphragm, and all positions at the annulus shown with a given number were included in the same subset for the annulus. The origin at the center of the diaphragm, which is indicated by an "O", was included in every DSMB diaphragm subset.

**Fig. 7:**
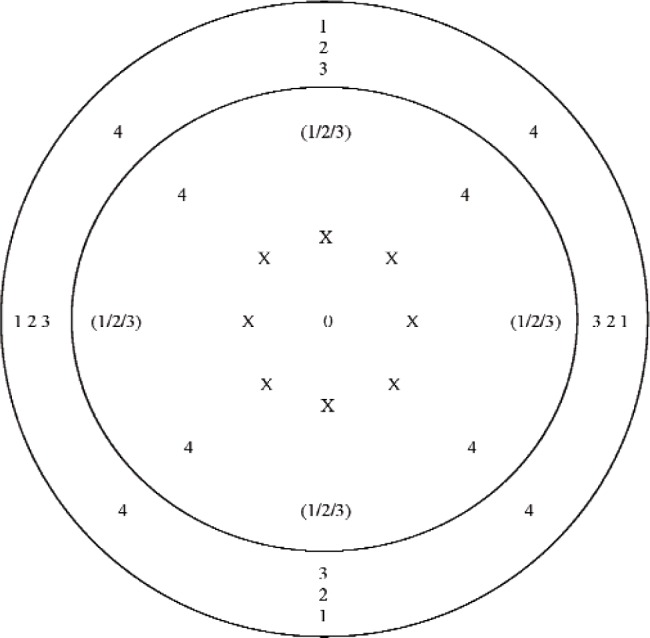
Approximate positions at the microphone annulus and diaphragm where data were acquired for direct measurement subsampling method C (DSMC). All positions at the diaphragm and the annulus shown with a given number were included in the same subset. The *x*–*y* origin at the center of the diaphragm, which is indicated by an “O”, was included in every subset. The four positions indicated by “(1/2/3)” at the diaphragm, were included in subsets 1, 2, and 3 of the four different subsets. An “X” indicates positions from the full data set that were not included in any DSMC subset. (Not to scale)

The third method determines the front cavity depth from the mean of five front cavity depths calculated from a subsampled data set comprising four positions at the annulus and five positions at the diaphragm. This method will be referred to as direct measurement sub-sampling method C (DSMC). Four of these depths are calculated from subsampled data by subtracting the *z*-axis coordinate of each of four positions measured at the diaphragm from the *z*-axis coordinate of the nearest corresponding position measured at the annulus. The fifth front cavity depth is the mean depth of the position located at the center of the diaphragm, which is determined by subtracting the *z*-axis coordinate of this position from the mean *z*-axis coordinate of the four positions measured at the annulus. The measurement positions included in the four subsets used are indicated in Fig. 7. All positions at the diaphragm and the annulus shown with a given number were included in the same subset. The position at the center of the diaphragm, which is indicated by an “O”, was included in every subset. The four positions indicated by “(1/2/3)” at the diaphragm, were included in subsets 1, 2, and 3 of the four different subsets. An “X” indicates positions from the full data set that were not included in any subset.

In general, the diaphragm and the plane defined at the high points of the annulus are not necessarily parallel to each other, or perpendicular to the *z* axis of measurement; i.e., *z* coordinates at this plane and at the diaphragm are functions of *x* and *y*. In an effort to account for such departures from ideal conditions, front cavity depths were calculated from the gage block measurement data by fitting the diaphragm data and the gage block data to two separate planes. The *z*-axis coordinate *z_d_* for the plane fit to the diaphragm data is given by

$$z_{d}(x,y)=A_{d}x+B_{d}y+C_{d}$$ (1)

where *A_d_* and *B_d_* are the coefficients that express the slope of the plane along the *x* and *y* axis respectively, and *C_d_* is the *z*-axis intercept at the center of the diaphragm where *x* and *y* equal zero. Likewise, the *z*-axis coordinate *z_g_* for the plane fit to the gage block data is given by

$$z_{g}(x,y)=A_{g}x+B_{g}y+C_{g}$$ (2)

where *A_g_* and *B_g_* are the coefficients that express the slope of the plane along the *x* and *y* axis respectively, and *C_g_* is the *z*-axis intercept for this plane. Fits to determine the coefficients and intercepts for both planes were done using the Regression Analysis Tool provided in the Analysis ToolPak available in Microsoft Excel 2002 SP-2. The front cavity depth *l_fc_* was determined by using the equation

$$l_{fc}=C_{g}-C_{d}-t$$ (3)

where *t* is the gage block thickness, which is 2.00005 mm ± 0.00012 mm (coverage factor *k* = 2). This thickness and expanded uncertainty are known from the calibration of this gage block and an estimate of the thermal effects from temperature variations measured in the laboratory. Plane fits were done with the full data sets, and with subsets of the full data sets corresponding to two different subsampling methods.

**Fig. 8:**
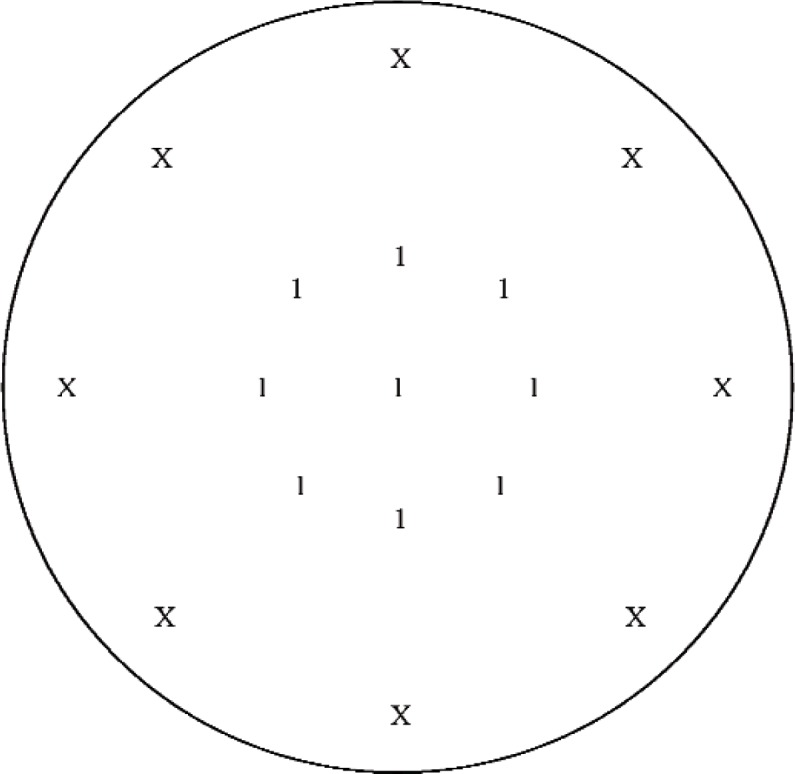
Approximate positions at the microphone diaphragm (portion viewed through the hole in the center of the gage block) where data were acquired for gage block measurement subsampling method D (GBSMD). The nine positions used from the full data set are shown with a “1”. An “X” indicates positions from the full data set that were not included in GBSMD. All of the data acquired for the full data set at the gage block surface were used for GBSMD. (Not to scale)

One subsampling method involved fitting all of the data acquired at the gage block surface, but only fitting nine of the positions measured at the surface of the diaphragm. These nine positions are shown with a “1” in Fig. 8. An “X” indicates positions from the full data set that were not included in the fit of this subset. This method will be referred to as gage block measurement subsampling method D (GBSMD).

**Fig. 9:**
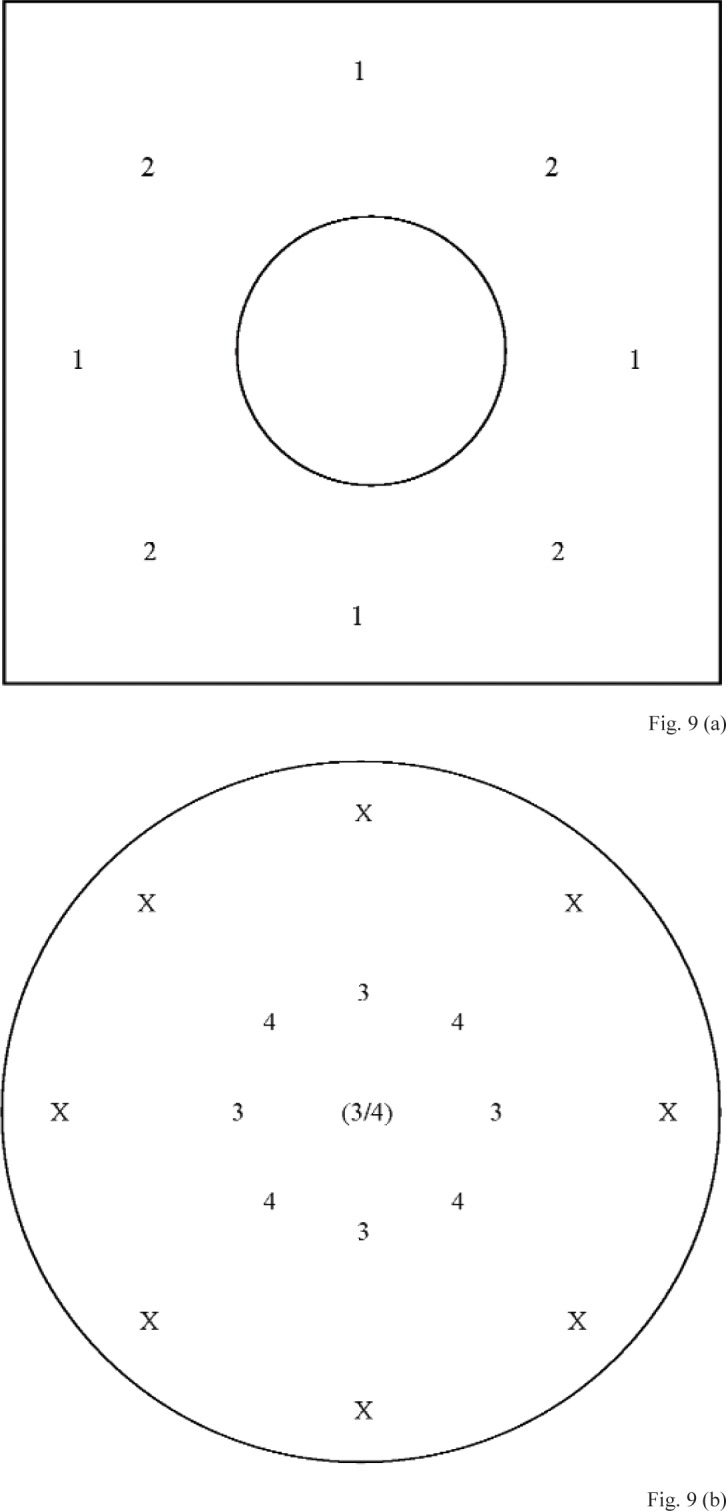
Approximate positions at the **(a)** gage block and **(b)** microphone diaphragm (portion viewed through the hole in the center of the gage block) where data were acquired for gage block measurement subsampling method E (GBSME). Positions shown with the same number were included in the same subset. The *x*–*y* origin at the center of the diaphragm, which is indicated by “(3/4)”, was included in both diaphragm subsets. An “X” indicates positions from the full data set that were not included in any subset. (Not to scale)

The second subsampling method involved fitting a subset comprising four positions at the gage block top surface, and fitting a subset comprising five positions measured at the diaphragm. This method will be referred to as gage block measurement subsampling method E (GBSME). Two different subsets were used for each surface to calculate four different *z*-axis intercepts, two for the diaphragm and two for the annulus. The measurement positions included in the subsets for the gage block surface are indicated in Fig. 9a, and for the diaphragm in Fig. 9b. Positions shown with the same number were included in the same subset. The origin at the diaphragm, which is indicated by “(3/4)”, was included in both diaphragm subsets. An “X” indicates positions from the full data set that were not included in any subset.

For each of the full data sets obtained using the D method, and for each of the full data sets obtained using the GB method, the *z*-axis coordinates measured at the diaphragm center at the start and end of each measurement set were used to check for drifts in the measurement system that could affect the data. For each full set of data, a difference was calculated by subtracting the *z*-axis coordinate measured at the diaphragm center initially from the *z*-axis coordinate measured at the diaphragm center after all other data were acquired. These differences were found to be in the range from −1.0 μm to 3.0 μm. The mean difference calculated from all data sets was determined to be 0.94 μm ± 0.29 μm (two experimental standard deviations of the mean), which indicates that on average, a slight drift that affected the data occurred in the measurement system during the course of measuring each data set. This drift was found to be due primarily to changes in the microscope system arising from thermal effects. Since the drift is considered to have occurred gradually over the time interval required to obtain a given full data set, and is modeled as a linear function of time, the front cavity depth is estimated to have a systematic error of 0.47 μm, which is half the mean difference calculated from all data sets. To compensate for this systematic error, all of the front cavity depths reported here have been adjusted by adding a correction equal to the negative of this error. The uncertainty in this correction is one of the components discussed in the Appendix on the uncertainty of front cavity depth measurement results.

## 5. Microscope Measurements on Reference Gage Blocks

To check the accuracy of the microscope measurement system and the GB method for front cavity depth measurement, this system and method were used to make several measurements on reference gage blocks. The thicknesses of these reference blocks were known from conventional dimensional calibrations and an estimate of the thermal effects from temperature variations measured in the laboratory. The expanded uncertainties of these thicknesses are at least an order of magnitude smaller than the expanded uncertainties of the front cavity depth measurement by the microscope system.

Gage blocks were wrung together to form stacks so that the thickness of the top block included the same nominal *z*-axis coordinate range that was used to measure the front cavity depth of a given microphone type. Stacks of different heights were prepared to cover the ranges applicable to measurements made with the microphone placed directly on the stage of the microscope and measurements made with the microphone on the spring-force fixture. In each case, the hole in the center of the top block was positioned in the *x*–*y* plane so that the upper surface of the block immediately below it was visible through the entire area of the hole. Data were acquired by focusing at the visible portion of this surface, and at the top block, to simulate the measurements made at a microphone diaphragm and at the surface of a gage block placed on the microphone annulus. The subsampling method used was equivalent to GBSMD, except the lower surface was a gage block instead of a microphone diaphragm. The thickness of the top block was determined from the data by fitting the data for the two surfaces independently to two separate planes, and then by subtracting the fitted *z*-axis intercept for the upper surface of the lower block from the fitted *z*-axis intercept for the upper surface of the top block. The same correction for systematic error was applied to these measurements as was applied to the microphone measurements (Sec. 4.)

Table 4 summarizes the results and setup details for the gage block thickness measurements done to simulate the microphone front cavity depth measurements made with the microphone placed directly on the stage of the microscope. Table 5 summarizes the results and setup details for the gage block thickness measurements done to simulate the microphone front cavity depth measurements made with the microphone placed on the spring-force fixture. Together, these tables show measurements by two different observers (#1 and #2), each of whom measured the top block thickness of each of the four different stacks. For the eight measurements (four by each observer), the difference between the measured values and the known values of the corresponding reference blocks ranged from −0.7 μm to 0.6 μm. The average of the absolute values of these differences was only 0.33 μm. These values are considerably smaller than the expanded uncertainties (of each of the eight measurements), which ranged from 1.9 μm to 2.3 μm, demonstrating that the microscope system for front cavity depth measurement performed well within these uncertainties.

## 6. Results and Discussion

### 6.1 Front Cavity Depth Measurements and Contact Force

**Fig. 10:**
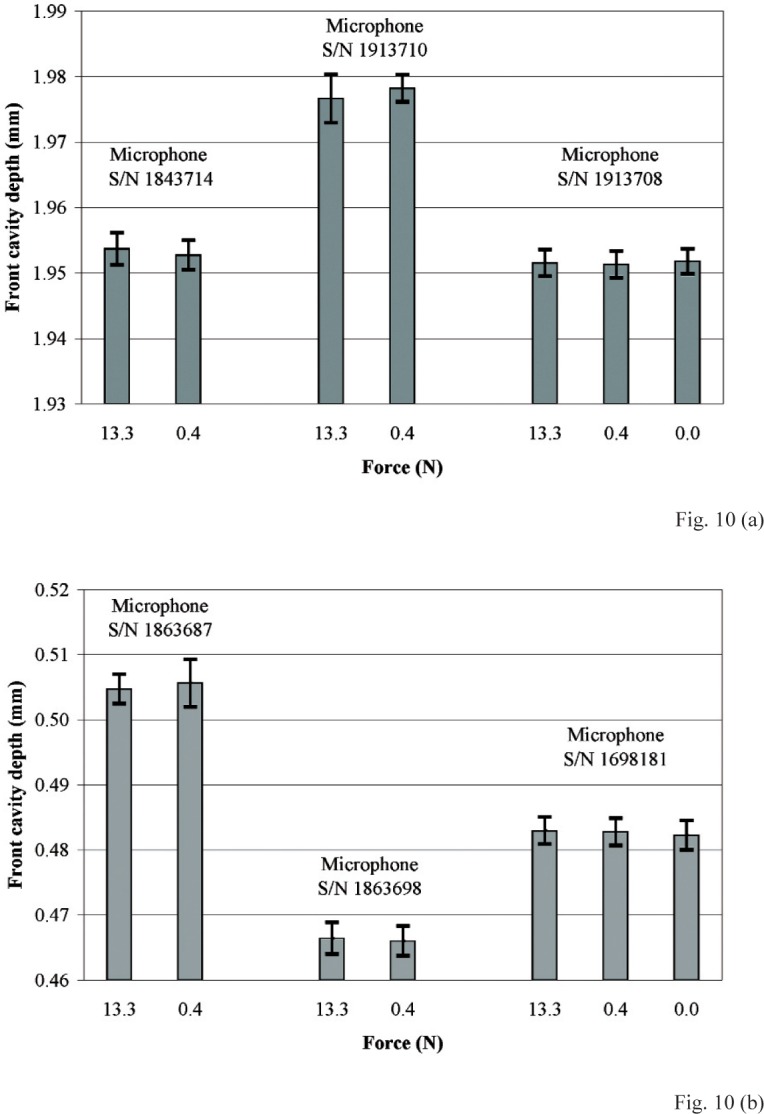
Comparison of front cavity depths measured with different compressive forces for three **(a)** IEC Type LS1P microphones, and for three **(b)** IEC Type LS2aP microphones. The upper and lower limits displayed with each mean indicate the expanded uncertainty (with coverage factor *k* = 2).

The spring-force fixture and the GB method were used with the microscope to measure front cavity depths for compressive contact forces 0 N, 0.4 N, and 13.3 N. This range of forces includes the range typically used during microphone calibration by the reciprocity method to maintain the annulus in contact with the mating surface of an acoustical plane-wave coupler. Plots of the measured front cavity depths at these applied forces given in Fig. 10a for LS1P microphones, and in Fig. 10b for LS2aP microphones, do not show any dependence of front cavity depth on applied force. For every microphone, the differences between front cavity depths measured at different forces lie well within the expanded uncertainties of measurement (with coverage factor *k* = 2) represented by the upper and lower limits displayed with each measurement in the plots. Therefore, these data are consistent with the commonly used, often implicit assumption that the highest portions of the annulus in contact with the plane surface of the gage block or an acoustical coupler are not deformed significantly by the applied compressive force.

### 6.2 Measurement Methods: Direct (D) vs. Gage Block (GB)

**Fig. 11:**
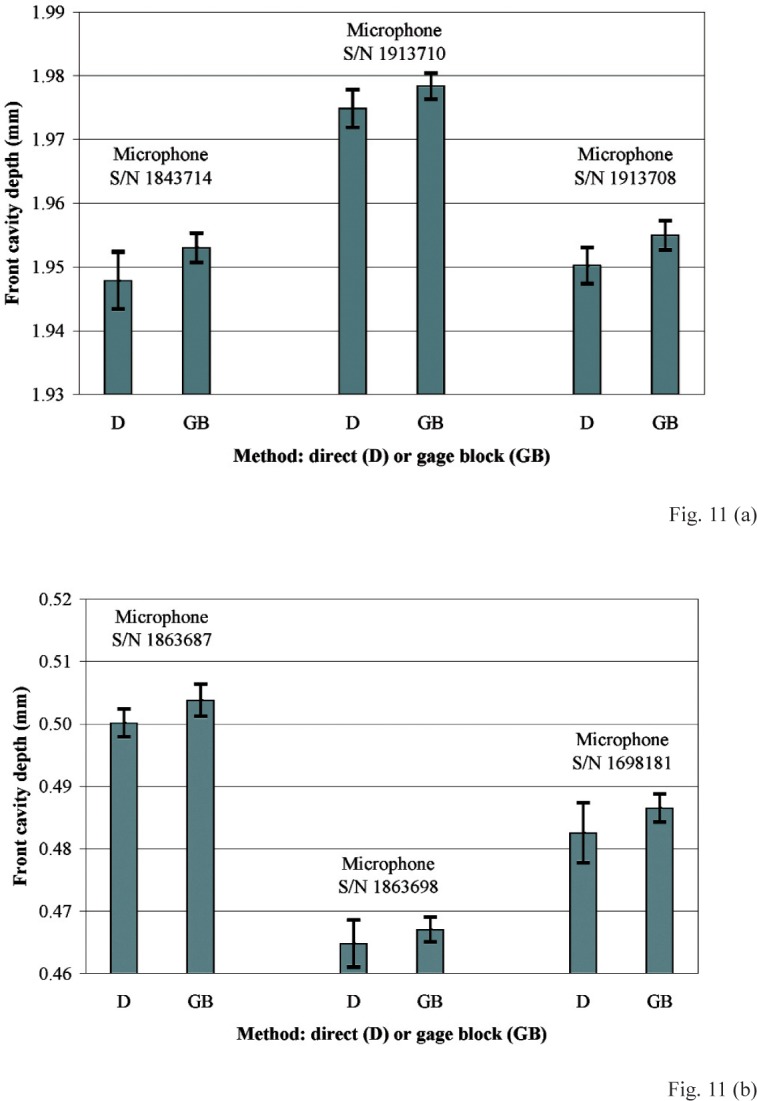
Comparison of front cavity depths measured using the direct (D) method with the front cavity depths measured using the gage block (GB) method for three **(a)** IEC Type LS1P microphones, and for three **(b)** IEC Type LS2aP microphones. The upper and lower limits displayed with each mean indicate the expanded uncertainty (with coverage factor *k* = 2).

In Fig. 11, front cavity depths determined from the full data set (33 positions) of measurements obtained using the direct (D) method are compared with front cavity depths determined from the full data set (25 positions) of measurements performed using the gage block method (GB). Figure 11a shows this comparison for each of the three LS1P microphones, and Fig. 11b shows this comparison for each of the three LS2aP microphones. For all six microphones, the GB front cavity depths are larger than the D front cavity depths. For the LS1P microphones (Fig. 11a), the front cavity depths determined using the GB method exceed the front cavity depths determined using the D method by 0.0035 mm to 0.0051 mm, and on average by 0.0045 mm. For the LS2aP microphones (Fig. 11b), the front cavity depths determined using the GB method exceed the front cavity depths determined using the D method by 0.0023 mm to 0.0040 mm, and on average by 0.0033 mm. These results are consistent with the provisional hypothesis that the inadequate spatial sampling used at the annulus in the D method does not necessarily include all of the highest points (of the ridges) of the annulus. Consequently, this sampling is inadequate to define the plane of contact between the highest points of the annulus and an acoustic coupler surface, so that the front cavity depth measurements performed using the D method tend to underestimate the front cavity depth during microphone calibration. In the GB method, however, the gage block provides a smooth plane surface that rests upon the high points of the annulus. This offers a better model than the D method of the contact between the annulus and the smooth plane surface of an acoustical coupler, and is considered to provide a better measure of the front cavity depth that forms a portion of the length of the coupling cavity (Fig. 2) used during microphone calibration.

For five of the six microphones, the front cavity depths determined from only 25 measurement positions per microphone using the GB method have smaller expanded uncertainties than the front cavity depths determined from 33 measurement positions per microphone using the D method. In this respect, the GB method is considered more useful than the D method, because the GB method usually achieves the better results, exhibiting smaller expanded uncertainties, while using fewer measurement positions.

### 6.3 Effects of Subsampling Methods

**Fig. 12:**
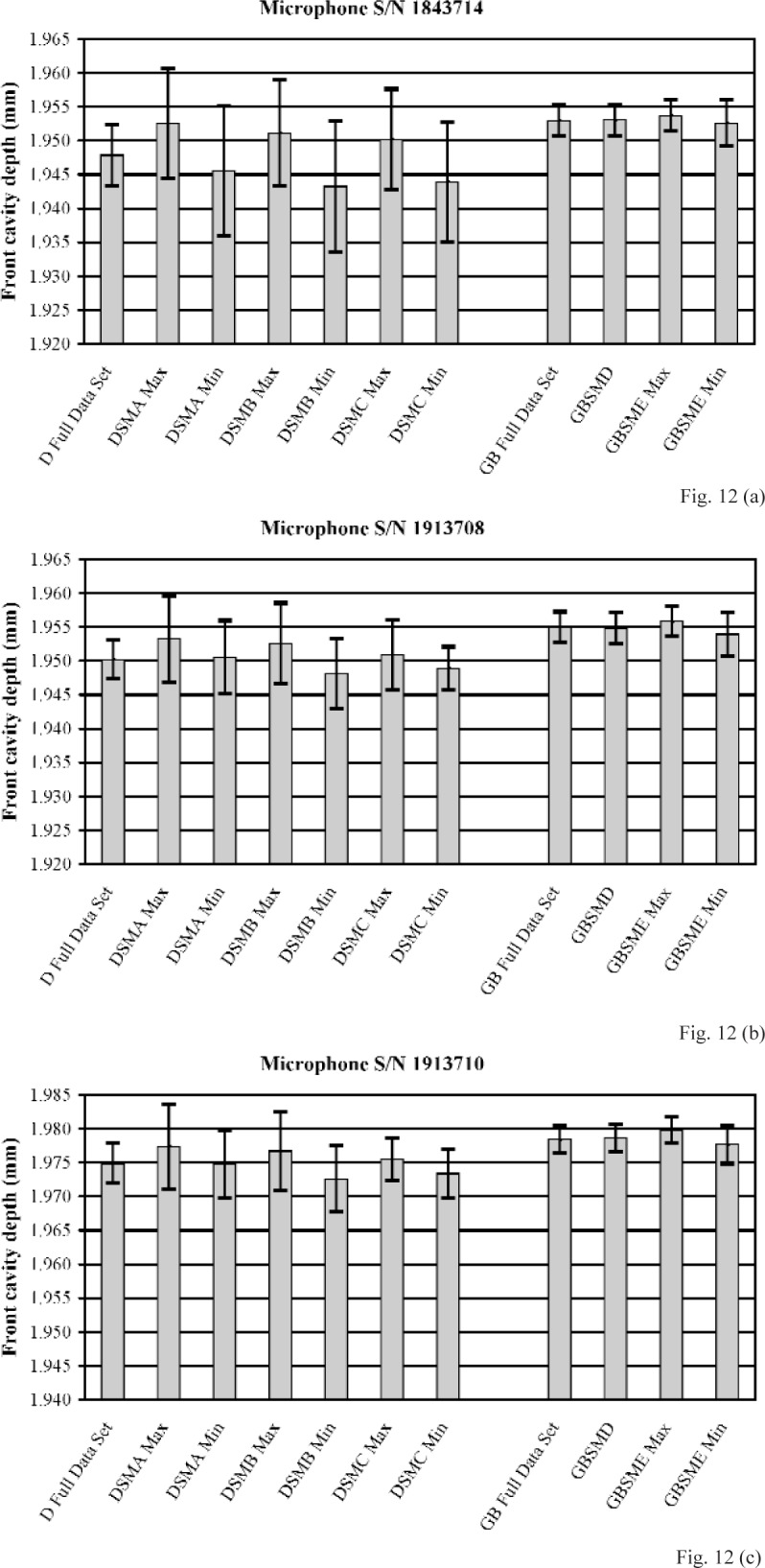
Comparison of the front cavity depths determined using the direct (D) method, the gage block (GB) method, and their various subsampling methods for LS1P microphones **(a)** S/N 1843714, **(b)** S/N 1913708, and **(c)** S/N 1913710. The upper and lower limits displayed with each mean indicate the expanded uncertainty (with coverage factor *k* = 2). The terms “Max” and “Min” refer to the maximum and minimum front cavity depths determined from the various subsets available for a given subsampling method.

**Fig. 13:**
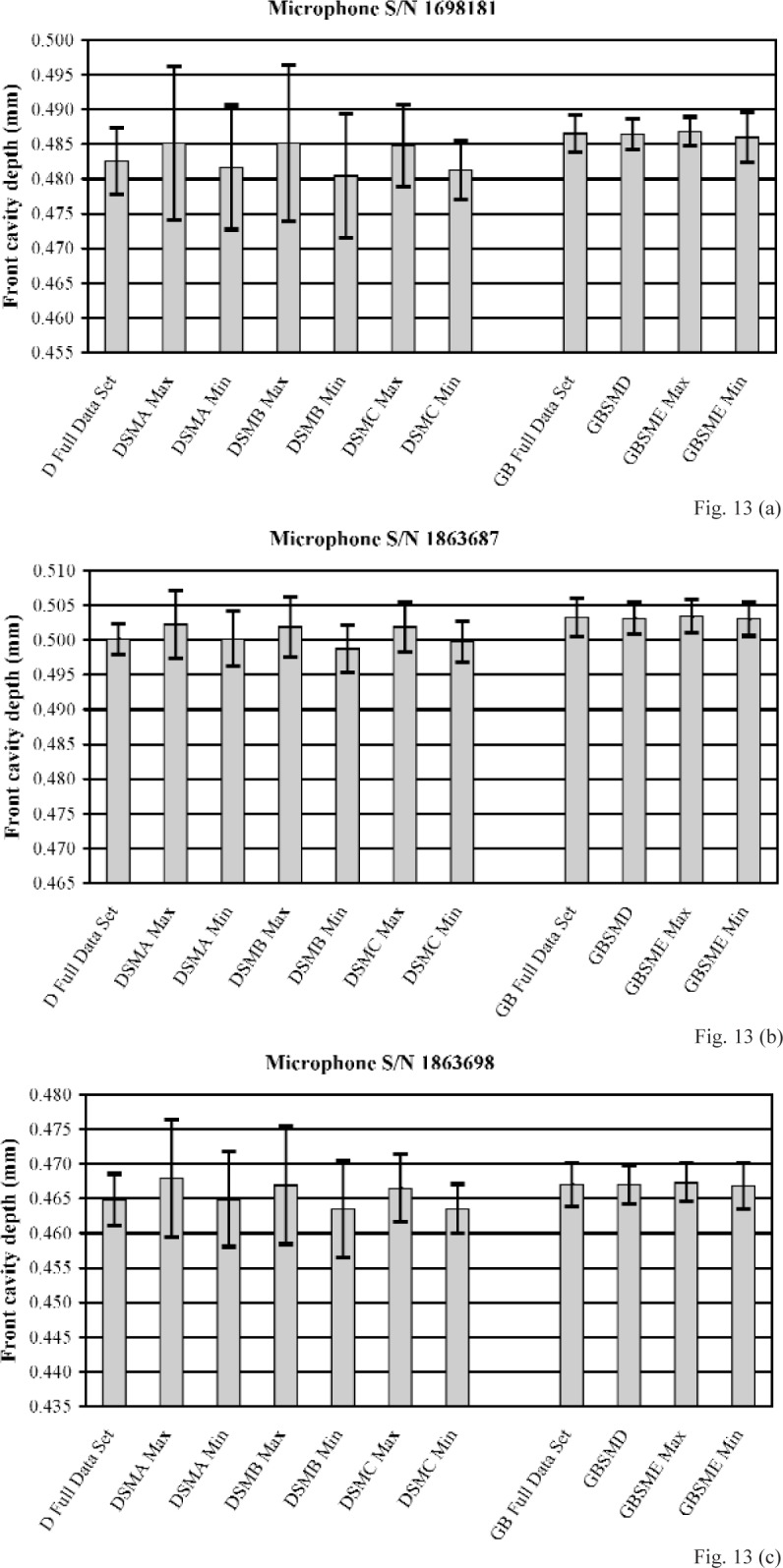
Comparison of the front cavity depths determined using the direct (D) method, the gage block (GB) method, and their various subsampling methods for LS2aP microphones **(a)** S/N 1698181, **(b)** S/N 1863687, and **(c)** S/N 1863698. The upper and lower limits displayed with each mean indicate the expanded uncertainty (with coverage factor *k* = 2). The terms “Max” and “Min” refer to the maximum and minimum front cavity depths determined from the various subsets available for a given subsampling method.

Figures 12 and 13 show front cavity depths determined from the full data set of measurements performed using the D method and from the subsampling of this set by methods DSMA, DSMB, and DSMC, and the front cavity depths determined from the full data set of measurements performed using the GB method and from the subsampling of this set by methods GBSMD and GBSME. Figure 12 shows these depths for the three LS1P microphones, and Fig. 13 shows these depths for the three LS2aP microphones. For each of the subsampling methods that was used to determine results from more than one data subset, the maximum and minimum values of the front cavity depths determined by the method are shown. In general, for the measurements performed using the D method, the front cavity depths determined with the subsampling methods display a wide range of values, and differ considerably from the front cavity depth determined from the full data set. The expanded uncertainties of these front cavity depths from the subsampling methods are shown (with coverage factor *k* = 2) as upper and lower limits in the figures. These uncertainties are often substantially (sometimes even a factor of two) larger than the expanded uncertainty of the front cavity depth from the full data set. For the measurements performed using the GB method, the front cavity depths determined with the GB subsampling methods differ relatively little from each other and from the front cavity depth determined from the full data set. Furthermore, the expanded uncertainties of these front cavity depths from the subsampling methods usually are comparable to, or only slightly larger than, the expanded uncertainty of the front cavity depth from the full data set. For each of all six microphones, even the front cavity depths determined by subsampling method GBSME, which uses data obtained at only 9 positions, agree more closely with the full data set result obtained using the GB method than does the front cavity depth determined from the full set of data obtained using the D method.. From these results, the subsampling methods performed using the gage block and plane-fitting data reduction techniques (GB subsampling methods) are considered more robust than the subsampling methods performed using the direct measurements and conventional data reduction techniques (D subsampling methods).

## 7. Summary and Conclusion

To achieve a high degree of accuracy in calibration of LS microphones using air-filled plane-wave couplers, the front cavity depths of individual microphones must be measured. An optical depth (*z*-axis) measuring microscope was used to examine several methods for measuring front cavity depth. These methods were then applied to three IEC Type LS1P and three IEC Type LS2aP microphones.

The basic capabilities of this microscope were demonstrated by using it to measure the thickness of the top block in each of four stacks of calibrated reference gage blocks that had been wrung together, so that the thickness of the top block included the same nominal *z*-axis range that was used to measure the front cavity depth of a given microphone type. For the eight measurements (four by each of two observers), the difference between the measured values and the known values of the corresponding reference blocks ranged from −0.7 μm to 0.6 μm. The average of the absolute values of these differences was only 0.33 μm. These values are considerably smaller than the expanded uncertainties (with coverage factor *k* = 2) that ranged from 1.9 μm to 2.3 μm for the various individual measurements, demonstrating that the microscope system for front cavity depth measurement performed well within these uncertainties.

The front cavity depth was measured over a range of applied compressive forces that includes the range typically used to maintain the annulus in contact with the mating surface of an acoustical coupler during pressure calibration of microphones by the reciprocity method. For every microphone, the differences between front cavity depths measured at different forces lie well within the expanded uncertainties of measurement. These results are consistent with the assumption that the highest portions of the annulus are not deformed significantly by the applied compressive force.

For each microphone, front cavity depths were determined from the full data set (33 positions) of measurements obtained using the direct (D) method, and also from the full data set (25 positions) of measurements performed using the gage block (GB) method. For five of the six microphones, the front cavity depths determined from only 25 measurement positions per microphone using the GB method show smaller expanded uncertainties than the front cavity depths determined from 33 measurement positions per microphone using the D method. In this respect, the GB method is preferable to the D method, because the GB method usually achieves better results with fewer measurement positions.

For each microphone, front cavity depths were also determined from the various subsampling methods. In general, the front cavity depths determined with the three different D subsampling methods display a wide range of values and differ considerably from the front cavity depth determined from the full data set of measurements performed using the D method. The expanded uncertainties of the front cavity depths from these D subsampling methods are often substantially (sometimes even a factor of two) larger than the expanded uncertainty of the front cavity depth from the full data set of measurements performed using the D method. The front cavity depths determined with two different GB subsampling methods differ relatively little from each other and from the front cavity depth determined from the full data set of measurements performed using the GB method. The expanded uncertainties of the front cavity depths calculated from these GB subsampling methods usually are comparable to, or only slightly larger than, the expanded uncertainty of the front cavity depth determined from the full data set of measurements performed using the GB method. For all microphones, the front cavity depths determined by the subsampling method GBSME, which uses only 9 measurement positions from the full data set obtained using the GB method, agree more closely with results from this full data set than do the front cavity depths determined from the full data set (33 positions) of measurements performed using the D method.

From these results, the GB subsampling methods provide measurements of front cavity depth that are in better agreement with measurements from more complete data sets, and provide smaller expanded uncertainties, than the D subsampling methods. In this sense, the subsampling methods performed using the gage block and plane fits are considered more robust than the subsampling methods performed using the direct measurements and conventional data reduction techniques, and are preferred for possible use in those evolving methods of microphone calibration using plane-wave acoustic couplers. Subsampling method GBSME is particularly convenient because it requires measurements at only nine positions, four at the gage block and five at the diaphragm.

Measurements of the front cavity depths of individual microphones by these methods produce uncertainties in these depths that are much smaller than the error that can result from the use of the nominal value of depth [1,2] for a microphone type, because the front cavity depth of a given microphone can differ substantially from this nominal value, yet remain within the generic standardized microphone type tolerance limits [1,2] on front cavity depths. A much smaller uncertainty in the front cavity depth leads to a much smaller uncertainty component in calibrations determining the pressure sensitivity level of microphones at high frequencies. For example, for an air-filled plane-wave coupling cavity formed by the cavity of a coupler 4.7 mm long and the front cavities of two IEC Type LS2aP microphones, a total error of ± 0.1 mm in the sum of the front cavity depths relative to the sum of generic standardized nominal values [1,2] would result in an error of ± 0.7 dB in microphone pressure sensitivity level at 25 kHz. This error alone would be at least 0.4 dB worse than the expanded uncertainty (with coverage factor *k* = 2) of primary calibration in pressure sensitivity level stated for this microphone type at this frequency by NIST and a number of other laboratories in a recent international key comparison [5] of best measurement capabilities. The front cavity depths of individual microphones are measured to achieve these capabilities. Performing these individual measurements by subsampling method GBSME or other methods that improve the error in the sum of the front cavity depths to ± 0.008 mm would result in an error from this component of only ± 0.06 dB in microphone pressure sensitivity level at 25 kHz. This represents an order of magnitude improvement over the result ± 0.7 dB obtained by using generic standardized [1,2] nominal values of microphone front cavity depth and tolerance limits.

## Figures and Tables

**Table 1:** Nominal *x*-axis and *y*-axis coordinates of the seventeen positions at the diaphragm, and the sixteen positions at the annulus, measured with the direct (D) method for IEC Type LS1P microphones. The *x*–*y* origin is at the center of the diaphragm.

Surface: diaphragm or annulus	Nominal *x*-axis coordinate (mm from diaphragm center)	Nominal *y*-axis coordinate (mm from diaphragm center)
diaphragm	0.000	0.000
diaphragm	0.000	8.101
diaphragm	0.000	−8.101
diaphragm	−8.101	0.000
diaphragm	8.101	0.000
diaphragm	5.728	5.728
diaphragm	5.728	−5.728
diaphragm	−5.728	−5.728
diaphragm	−5.728	5.728
diaphragm	0.000	2.749
diaphragm	0.000	−2.749
diaphragm	−2.749	0.000
diaphragm	2.749	0.000
diaphragm	1.944	1.944
diaphragm	1.944	−1.944
diaphragm	−1.944	−1.944
diaphragm	−1.944	1.944
annulus	0.000	11.284
annulus	0.000	10.592
annulus	0.000	9.900
annulus	0.000	−9.900
annulus	0.000	−10.592
annulus	0.000	−11.284
annulus	−11.284	0.000
annulus	−10.592	0.000
annulus	−9.900	0.000
annulus	9.900	0.000
annulus	10.592	0.000
annulus	11.284	0.000
annulus	7.490	7.490
annulus	7.490	−7.490
annulus	−7.490	−7.490
annulus	−7.490	7.490

**Table 2:** Nominal *x*-axis and *y*-axis coordinates of the seventeen positions at the diaphragm, and the sixteen positions at the annulus, measured with the direct (D) method for IEC Type LS2aP microphones. The *x*–*y* origin is at the center of the diaphragm.

Surface: diaphragm or annulus	Nominal *x*-axis coordinate (mm from diaphragm center)	Nominal *y*-axis coordinate (mm from diaphragm center)
diaphragm	0.000	0.000
diaphragm	0.000	3.750
diaphragm	0.000	−3.750
diaphragm	−3.750	0.000
diaphragm	3.750	0.000
diaphragm	2.652	2.652
diaphragm	2.652	−2.652
diaphragm	−2.652	−2.652
diaphragm	−2.652	2.652
diaphragm	0.000	2.749
diaphragm	0.000	−2.749
diaphragm	−2.749	0.000
diaphragm	2.749	0.000
diaphragm	1.944	1.944
diaphragm	1.944	−1.944
diaphragm	−1.944	−1.944
diaphragm	−1.944	1.944
annulus	0.000	6.225
annulus	0.000	5.625
annulus	0.000	5.025
annulus	0.000	−5.025
annulus	0.000	−5.625
annulus	0.000	−6.225
annulus	−6.225	0.000
annulus	−5.625	0.000
annulus	−5.025	0.000
annulus	5.025	0.000
annulus	5.625	0.000
annulus	6.225	0.000
annulus	3.978	3.978
annulus	3.978	−3.978
annulus	−3.978	−3.978
annulus	−3.978	3.978

**Table 3:** Nominal *x*-axis and *y*-axis coordinates of the seventeen positions at the diaphragm, and the eight positions at the upper surface of the gage block, measured with the gage block (GB) method for IEC Type LS1P and IEC Type LS2aP microphones. The *x*–*y* origin is at the center of the diaphragm.

Surface: diaphragm or gage block	Nominal *x*-axis coordinate (mm from diaphragm center)	Nominal *y*-axis coordinate (mm from diaphragm center)
diaphragm	0.000	0.000
diaphragm	0.000	2.749
diaphragm	0.000	−2.749
diaphragm	−2.749	0.000
diaphragm	2.749	0.000
diaphragm	1.944	1.944
diaphragm	1.944	−1.944
diaphragm	−1.944	−1.944
diaphragm	−1.944	1.944
diaphragm	0.000	1.375
diaphragm	0.000	−1.375
diaphragm	−1.375	0.000
diaphragm	1.375	0.000
diaphragm	0.972	0.972
diaphragm	0.972	−0.972
diaphragm	−0.972	−0.972
diaphragm	−0.972	0.972
gage block	0.000	10.592
gage block	0.000	−10.592
gage block	−10.592	0.000
gage block	−10.592	0.000
gage block	7.490	7.490
gage block	7.490	−7.490
gage block	−7.490	−7.490
gage block	−7.490	7.490

**Table 4:** Summary of the results and the setup details from the reference gage block thickness measurements done to simulate the microphone front cavity depth measurements made with the microphone placed directly on the stage of the microscope. The nominal *z*-axis coordinates are given in millimeters relative to the top surface of the microscope stage.

Microphone type	LS1P	LS2aP
Nominal *z*-axis coordinate of 2 mm gage block upper surface for front cavity depth measurements	21.00	14.00
Nominal *z*-axis coordinate of microphone diaphragm for front cavity depth measurements	17.05	11.50
Nominal *z*-axis coordinate of top gage block upper surface for gage block thickness measurements	21.7^a^	14.000
Nominal *z*-axis coordinate of lower gage block upper surface for gage block thickness measurements	16.7^a^	10.000
Top gage block thickness (mm) and ± expanded uncertainty (mm) with coverage factor *k* = 2	5.00011 ± 0.00020	4.00005 ± 0.00017
Measured thickness of top gage block for observer #1 (mm) and ± expanded uncertainty (mm) with coverage factor *k* = 2	5.0000 ± 0.0022	4.0000 ± 0.0019
Measured thickness of top gage block for observer #2 (mm) and ± expanded uncertainty (mm) with coverage factor *k* = 2	4.9998 ± 0.0023	4.0006 ± 0.0021

a Gage block stack was wrung on top of a 12.7 mm thick platen placed directly on the microscope stage.

**Table 5:** Summary of the results and the setup details from the reference gage block thickness measurements done to simulate the microphone front cavity depth measurements made with the microphone placed on the spring-force fixture. The nominal *z*-axis coordinates are given in millimeters relative to the top surface of the microscope stage.

Microphone type	LS1P	LS2aP
Nominal *z*-axis coordinate of 2 mm gage block upper surface for front cavity depth measurements	34.60	27.60
Nominal *z*-axis coordinate of microphone diaphragm for front cavity depth measurements	30.65	25.10
Nominal *z*-axis coordinate of top gage block upper surface for gage block thickness measurements	35.000	28.000
Nominal *z*-axis coordinate of lower gage block upper surface for gage block thickness measurements	30.000	25.000
Top gage block thickness (mm) and ± expanded uncertainty (mm) with coverage factor *k* = 2	5.00011 ± 0.00020	3.00005 ± 0.00014
Measured thickness of top gage block for observer #1 (mm) and ± expanded uncertainty (mm) with coverage factor *k* = 2	5.0003 ± 0.0019	2.9994 ± 0.0021
Measured thickness of top gage block for observer #2 (mm) and ± expanded uncertainty (mm) with coverage factor *k* = 2	5.0000 ± 0.0020	2.9994 ± 0.0021

**Table 6:** Summary of uncertainties of the microphone front cavity depth measurement results obtained with the GB method. With the exception of the uncertainty due to the correction for systematic error, numbers in the table apply only to this specific example, the uncertainty calculation for the LS2aP microphone with serial number 1863687.

Source of uncertainty	Standard uncertainty (μm)
Standard error in plane fit of *C_g_*, the *z*-axis intercept of the gage block plane (Type A)	0.62
Standard error in plane fit of *C_d_*, the *z*-axis intercept of the diaphragm plane (Type A)	0.18
Correction for systematic error, applied to compensate for observed drift in measurement system (Type A)	0.87
Centering of gage block over *x*-axis center of diaphragm (Type B)	0.70
Centering of gage block over *y*-axis center of diaphragm (Type B)	0.14
Gage block thickness due to uncertainty in its calibration and thermal effects in laboratory (Type B)	0.06
Combined standard uncertainty	1.3
Expanded (coverage factor *k* = 2) uncertainty	2.6

**Table 7:** Summary of uncertainties of the microphone front cavity depth measurement results obtained with the D method. With the exception of the uncertainty due to the correction for systematic error, numbers in the table apply only to this specific example, the uncertainty calculation for the LS2aP microphone with serial number 1698181.

Source of uncertainty	Standard uncertainty (μm)
Standard deviation of mean *z*-axis coordinate for annulus (Type A)	2.14
Standard deviation of mean *z*-axis coordinate for diaphragm (Type A)	0.64
Correction for systematic error, applied to compensate for observed drift in measurement system (Type A)	0.87
Combined standard uncertainty	2.4
Expanded (coverage factor *k* = 2) uncertainty	4.8

## Footnotes

1 This sensitivity is the amplitude ratio of microphone open-circuit (microphone output terminals electrically unloaded so that negligible current flows) output voltage to the sound pressure uniformly distributed over the diaphragm surface. This sensitivity is used to measure the sound pressure in spatially uniform sound fields, for example, in the calibration of widely used sound calibrators, as well as calibration of supra-aural and insert audiometric and other earphones in acoustic couplers. Sensitivity is usually expressed as the ratio of SI derived units V/Pa.

2 The pressure sensitivity level (pressure response level) of a microphone is expressed in decibels (dB) with the value of the reference quantity 1 V/Pa.

3 Certain commercial equipment, instruments, or materials are identified in this paper to foster understanding. Such identification does not imply recommendation or endorsement by the National Institute of Standards and Technology, nor does it imply that the materials or equipment identified are necessarily the best available for the purpose.

## 8. Appendix—Uncertainties of Microphone Front Cavity Depth and Gage Block Thickness Measurement Results

Expanded uncertainties of the measured microphone front cavity depths and gage block thicknesses were estimated by applying recognized guidelines for evaluating and expressing uncertainties [7].

For the microphone front cavity depth measurement results obtained with the GB method, the components of standard uncertainty are summarized in Table 6, which is shown as an example. This table contains numbers that apply specifically to the uncertainty calculation for the LS2aP microphone serial number 1863687. Two Type A standard uncertainties, equal to the standard errors in the Excel-fitted values of the *z*-axis intercepts *C_d_* in Eq. (1) and *C_g_* in Eq. (2), are included. The uncertainty in the correction for systematic error is also included. This Type A standard uncertainty is equal to the experimental standard deviation (0.87 μm) of the thirty-five differences used to calculate the mean drift (Sec. 4) from which the correction was derived. Two Type B standard uncertainties are included to take account of the effect of the uncertainty in setting the *x*–*y* coordinate system origin at the center of the diaphragm with the gage block in place on the annulus. To estimate the uncertainty of the front cavity depth due to an offset in the *x*-axis origin, the difference between the coefficients *A_d_* in Eq. (1) and *A_g_* in Eq. (2) was multiplied by the standard uncertainty in this offset, which was determined by assuming a symmetric rectangular probability distribution with estimated upper and lower bounds of ± 1.00 mm. Since the standard deviation of such a distribution [7] is one-half the width of the distribution multiplied by a factor of 0.577, the standard uncertainty in the origin offset is therefore equal to 0.577 mm. A similar approach was used to estimate the standard uncertainty of the front cavity depth due to a *y*-axis origin offset. The difference between the coefficients *B_d_* in Eq. (1) and *B_g_* in Eq. (2) was multiplied by the standard uncertainty in the *y*-axis offset, which was also estimated to be 0.577 mm. Another Type B standard uncertainty due to the uncertainty in the gage block thickness was estimated to be 0.06 μm. This estimate was obtained from the uncertainty of the gage block calibration and an estimate of thermal effects due to changes in laboratory temperature during the measurements of the front cavity depth.

For the reference gage block thickness measurement results, the uncertainty was calculated in the same manner, with three exceptions. First, because the gage block thickness is measured as the end result, and is not used as a known constant in Eq. (3) as it is when determining the front cavity depth, the uncertainty in the gage block thickness due to its own calibration and an estimate of thermal effects is not included. The remaining exceptions are the two Type B uncertainties due to the uncertainties in the *x*-axis and *y*-axis centering of the gage block over the diaphragm center, which are not relevant to the gage block thickness measurement.

For the microphone front cavity depth measurement results obtained with the D method, the components of standard uncertainty are summarized in Table 7, which is shown as an example. This table contains numbers that apply specifically to the uncertainty calculation for the LS2aP microphone with serial number 1698181. Two Type A standard uncertainties, one equal to the standard deviation of the mean *z*-axis coordinate determined for the annulus, and another equal to the standard deviation of the mean *z*-axis coordinate determined for the diaphragm, are included. Both of these Type A standard uncertainties were included in the uncertainty calculations for the results obtained with DSMA, DSMB, and the full data sets. However, since the z-axis coordinate of the diaphragm for DSMA was derived from only a single measurement that was performed at the center of the diaphragm, it was necessary to estimate a Type B standard uncertainty for this single measurement to use instead of a Type A derived from several measurements. For DSMC, only a single Type A standard uncertainty was calculated since this method involved calculating the mean of several differences in *z*-axis coordinates. This uncertainty is equal to the standard deviation of the mean difference determined from the set of differences measured between the annulus and diaphragm. For all of the direct measurement methods, the Type A standard uncertainty in the correction for systematic error was also included in the uncertainty calculations.
